# Determination of the high-pressure–temperature phase of LiMnPO_4_ energised by battery applications

**DOI:** 10.1007/s10853-026-13008-z

**Published:** 2026-06-07

**Authors:** Joshua A. H. Littleton, Andrew J. M. Evans, Julia Neukampf, Tara R. McElhinney, Simon A. Hunt

**Affiliations:** 1 Department of Materials, University of Manchester, Manchester, M13 9PL UK; 2 Department of Earth and Environmental Sciences, University of Manchester, Manchester, M13 9PY UK

## Abstract

Olivine-structured lithium-metal-phosphates (α-LiMPO_4_, where M = Fe, Ni, Co, Mn) are established or prospective cathode materials for lithium-ion batteries. At high pressures (P, in gigapascal; GPa) and temperatures (T, in Celsius; °C), the electrochemically active α-LiFePO_4_, α-LiNiPO_4_, and α-LiCoPO_4_ undergo a solid-state phase transition to an electrochemically less active or inactive *Cmcm* (β) orthorhombic structure. This phase delineation in P–T space is significant because it differentiates between α and β phases and constrains boundary conditions for the synthesis of these cathode materials. To date, pressure–temperature phase diagrams showing this delineation in P–T space, either via theoretical investigations or experimental results, have not been reported for any of the LiMPO_4_ cathode materials. In this work, we conducted experiments on α-LiMnPO_4_ up to 8 GPa and 1113 °C in a large-volume hydraulic press. Using ex situ X-ray diffraction and Raman spectroscopy, we determined the high P–T phase to be a β structure (*a* = 5.5465(3) Å; *b* = 8.4110(6) Å; *c* = 6.2990(4) Å) and reported the first phase diagram up to 9 GPa and 1200 °C. A linear Clapeyron relation was determined to be − 3.15 MPa/°C and predicted a room-temperature α → β transition at ~ 8.2 GPa.

**Supplementary Information:**

The online version contains supplementary material available at 10.1007/s10853-026-13008-z.

## Introduction

Lithium manganese (II) phosphate, or lithiophilite (Lhp, LiMnPO_4_), is the manganese end member in a solid solution with triphylite (Trp, LiFePO_4_), as the iron end member. The Lhp-Trp solid solution system is isostructural with olivine [1] and occurs naturally, most commonly as accessory minerals in geologically rare lithium-caesium-tantalum pegmatites [2, 3]. These pegmatites are the source for approximately 60% of global lithium production and are exploit primarily from spodumene, although other Li-rich phases including lepidolite and petalite are of economic importance [3–5]. The global Li demand is projected to outpace supply by 2030 [6], and this predicted deficit of Li is driving both the development of novel technologies and exploration of new and unconventional resources, including sedimentary-hosted deposits and geothermal brines [7–9]. For instance, Reich et al. [10] developed a direct chemical extraction process to delithiate Trp from geothermal brines that, under ideal conditions, achieved considerably high recovery rates of up to 99% within one extraction cycle. At an industrial scale, this promising approach could make Lhp and Trp economically viable Li-bearing minerals.

The commercial and industrial applications of Li are diverse, including metallic Li as a high-capacity hydrogen-storage material for hydrogen fuel cells [11], liquid Li as a coolant and tritium breeder material in thermonuclear fusion reactors [12], low-density Li alloys for aerospace and defence sectors [13], and Li compounds as a combustible fuel within propellants and propulsion systems [14]. The most recognised and widely used application of Li currently driving demand is lithium-ion batteries for consumer electronics [6–8], which have a significant foothold in commercial markets due to having a higher energy density and efficiency, lower self-discharge rate, and longer life cycle compared to other rechargeable batteries. Lithium-ion batteries charge/discharge via intercalation of Li^1+^ within the cathode and anode materials and involve the transport of the ions in a liquid electrolyte through a porous membrane that separates and mechanically isolates the electrodes [15]. The performance and properties of a lithium-ion battery cell depend on these four main components, and thus, the materials that comprise each component must be carefully considered. Olivine-structured phosphates, such as Lhp and Trp, as cathode materials were first investigated by Padhi et al. [16] who showed that the high specific capacity, flat open-circuit voltage, and good resistance to charge/discharge cycling indicated a promising battery cathode. These advantageous electrochemical properties were complemented by relatively low manufacturing costs, low toxicity, reduced environmental footprint, and high safety levels compared to other cathode materials [16, 17]. Since then, these materials have garnered more discussion and interest as synthesis, (de)lithiation, and performance manipulation (i.e. doping/additives, surface coating) techniques improved over the last two decades [18–20]. Moreover, olivine-structured phosphates, namely Trp, are one of several cathode materials currently used in batteries for electrical vehicles and are expected to experience continued market volume growth in the future [21, 22].

Recently, theoretical and experimental works on lithium-ion battery component materials have expanded the focus to high-pressure (P) and low-to-high temperature (T) conditions for two main reasons: i) to assess the stability of existing materials at non-ambient conditions for specialised applications [23–30] and ii) to synthesise and characterise new stable phases that may have more advantageous electrochemical properties [31–35]. Trp, LiNiPO_4_, and LiCoPO_4_, two additional olivine-structured phosphate and candidate cathode materials, all underwent a structural transformation from the orthorhombic *Pnma* olivine (α) structure to an orthorhombic *Cmcm* (β) structure [36–38] with increasing P and T. The α structure consists of a distorted hexagonal closed-packed framework array of O^2−^ that contains two octahedral (M1 and M2) sites for Li^1+^ (M1) and M^2+^ (M2; M^2+^  = Fe^2+^, Ni^2+^, Co^2+^, Mn^2+^) and a tetrahedral site for P^5+^ [20]. In this arrangement, the Li^1+^ occupying the edge-sharing octahedral M1 sites forms collinear chains in all crystallographic directions; however, because the Li–Li distance is shortest, one-dimensional ionic diffusion (i.e. hopping) along the [0 1 0] direction is the predominant pathway [20, 38, 40]. It is due to the stability of the PO_4_^3−^ tetrahedron that prevents significant structural contraction and collapse during (de)lithiation [41]. While the α → β transition is accompanied by a change in crystal symmetry, the tetrahedral and octahedral sites are retained. In the β phase, an FePO_4_^−1^ subarray is arranged like *Cmcm* structured FeSO_4_, where octahedral M^2+^ sites share edges with two equivalent octahedrons and corners with PO_4_^3−^ tetrahedrons, with Li^1+^ occupying remaining octahedral voids [42]. Since the ambient structures are alike and the ionic radii of Mn^2+^  > Fe^2+^  > Co^2+^  > Ni^2+^ are similar, Lhp may also undergo an α → β transformation at high P and T.

Assat and Manthiram [43] developed a low T (< 300 °C) and P (3 MPa) methodology using rapid microwave-assisted solvothermal synthesis to form the β structure of LiMnPO_4_, and this was the first work to identify a non-olivine structure for the compound. However, after a small increase in T (~ 175 °C) beyond that required for synthesis, a complete β → α transformation occurred. Compared to α, β-LiMPO_4_ (M = Fe, Ni, Co, or Mn) has reduced to inactive electrochemical performance because of decreased mobility of Li^1+^ for (de)lithiation [38, 39, 43]. Density functional theory-based molecular dynamic calculations showed that in the β phase the Li–Li distance was larger compared to the α phase, where the latter also has a higher degree of collinearity of Li along the lattice, and the Li–O distance was shortened to a comparable distance as in Li_2_O [38]. As a result, hopping of Li^1+^ through the Li sites of the β phase lattice is considerably diminished, if not completely inhibited, compared to the α phase. Thus, the α-β phase boundary is crucial to determine because it delineates electrochemically active and inactive phases and constrains synthesis processes and conditions of olivine-structured phosphate cathode materials. While a non-olivine-structured β-LiMnPO_4_ has been observed, the stability field of the β phase in P–T space has not been investigated nor has it been shown that the high P phase of LiMnPO_4_ has a *Cmcm* structure.

In this work, we report, to the best of our knowledge, the first P–T phase diagram delineating the onset of a high P phase of LiMnPO_4_ and determination of its solid-state structure. High P–T hot-pressing synthesis experiments were carried out up to 8 GPa and 1113 °C using a Walker-style multi-anvil apparatus. Phase structures were determined using Raman spectroscopy and X-ray diffraction (XRD).

## Methods

### High-pressure experiments

#### High-pressure cell design

**Figure 1:**
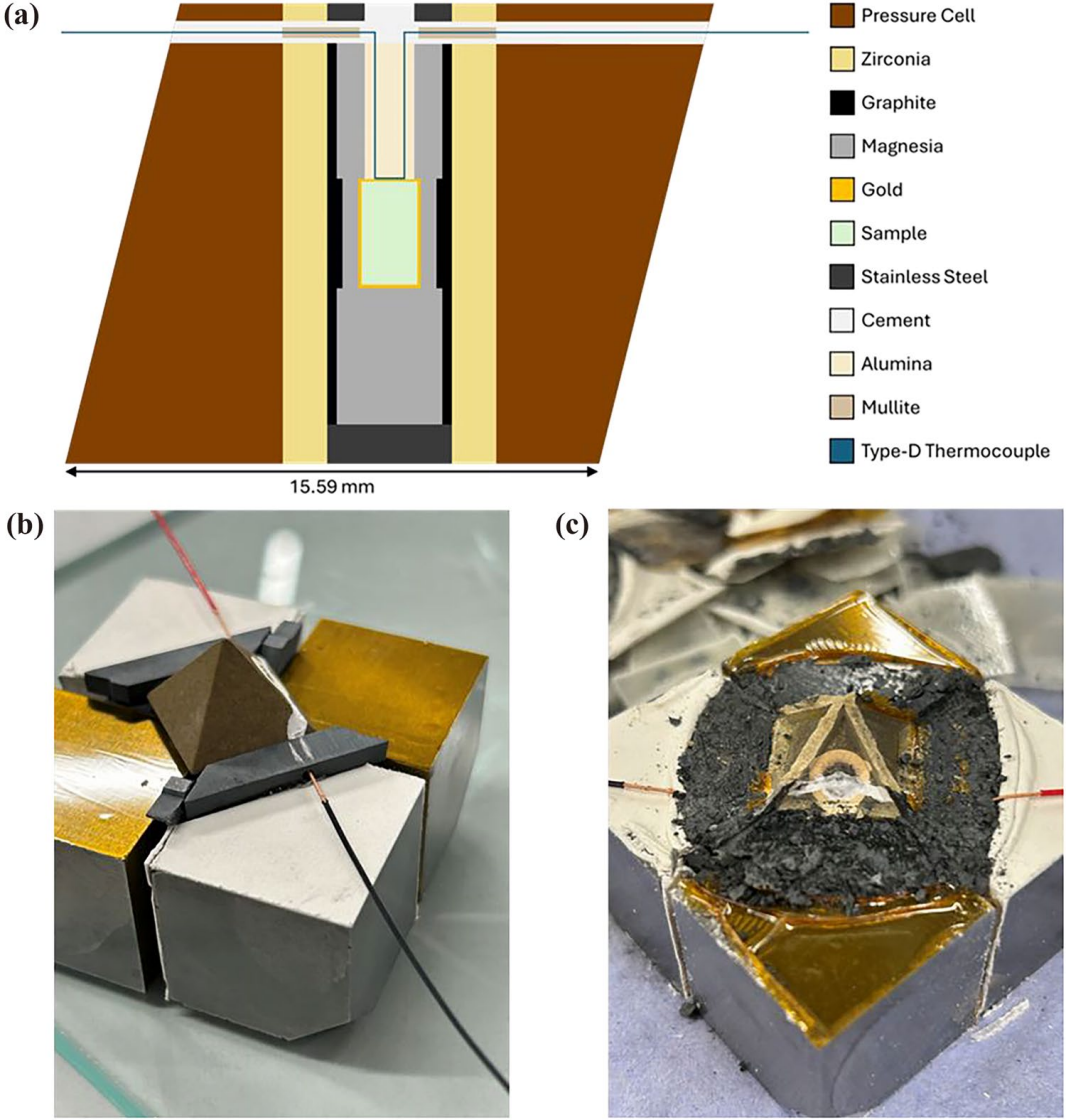
**a** An illustration depicting the cross-section of the high-pressure cell design used to conduct hot-pressing experiments. In three dimensions, the high-pressure cell is a regular octahedron with edge lengths of 18 mm. **b** A fully assembled high-pressure cell sitting in four truncated tungsten-carbide anvils. The thermocouple wires extruding the high-pressure cell are wound with copper wire and threaded through holes drilled into pyrophyllite gaskets. The anvils are electrically isolated using yellow insulation tape and white cardboard paper. Four more anvils with gaskets and electrical insulation are placed directly on top of this assembly prior to conducting hot-pressing experiments. **c** A post-experiment high-pressure cell. The triangular face shown is looking top-down at the high-pressure cell in Fig. 1a. The tops of the zirconia annulus, crescent-shaped stainless-steel disc, and alumina-based cement are visible.

As illustrated in Fig. 1a, the samples were housed in a low-density chromia-doped magnesia octahedron high P cell with 18-mm edge lengths. A cylindrical annulus of zirconia (14.70 mm × 6.85 mm) with a 4.00-mm-diameter hole was used as thermal insulation that surrounded a graphite step furnace lengthwise. The step furnace consisted of two longer (4.35 mm) and thinner-walled cylindrical graphite sleeves (4.00 mm outer diameter and 3.40 mm inner diameter) that were stacked above and below a shorter (3.50 mm) and thicker-walled (4.00 mm outer diameter and 3.00 mm inner diameter) sleeve. LiMnPO_4_ powder (99.9% purity) was purchased from Tokyo Chemical Industry UK Ltd. and packed in a cylindrically shaped gold foil capsule (3.50 mm × 2.00 mm) inside of high-density magnesia inserted into the middle graphite sleeve. High-density magnesia was inserted into the other two sleeves, with the magnesia of the upper sleeve bearing a single hole (1.60 mm diameter) for placement of a Type-D thermocouple (W/3%Re-W/25%Re) inside of a four-hole alumina tube (4.90 mm length). The ends of the step furnace were capped by a stainless-steel disc at the bottom and crescent-shaped mild-steel piece at the top to allow positioning of the thermocouple wires inside of two grooves cut into zirconia and the octahedron (Fig. 1b). The wires inside of the high P cell were sheathed by mullite tubes and an alumina-based cement mixture (Feuerfestkitt, Morgan Advanced Materials), where the latter also served to fill the gap around the steel crescent and the empty space in the grooves that allowed extrusion of the wires out of the cell.

#### High-pressure apparatus

**Figure 2:**
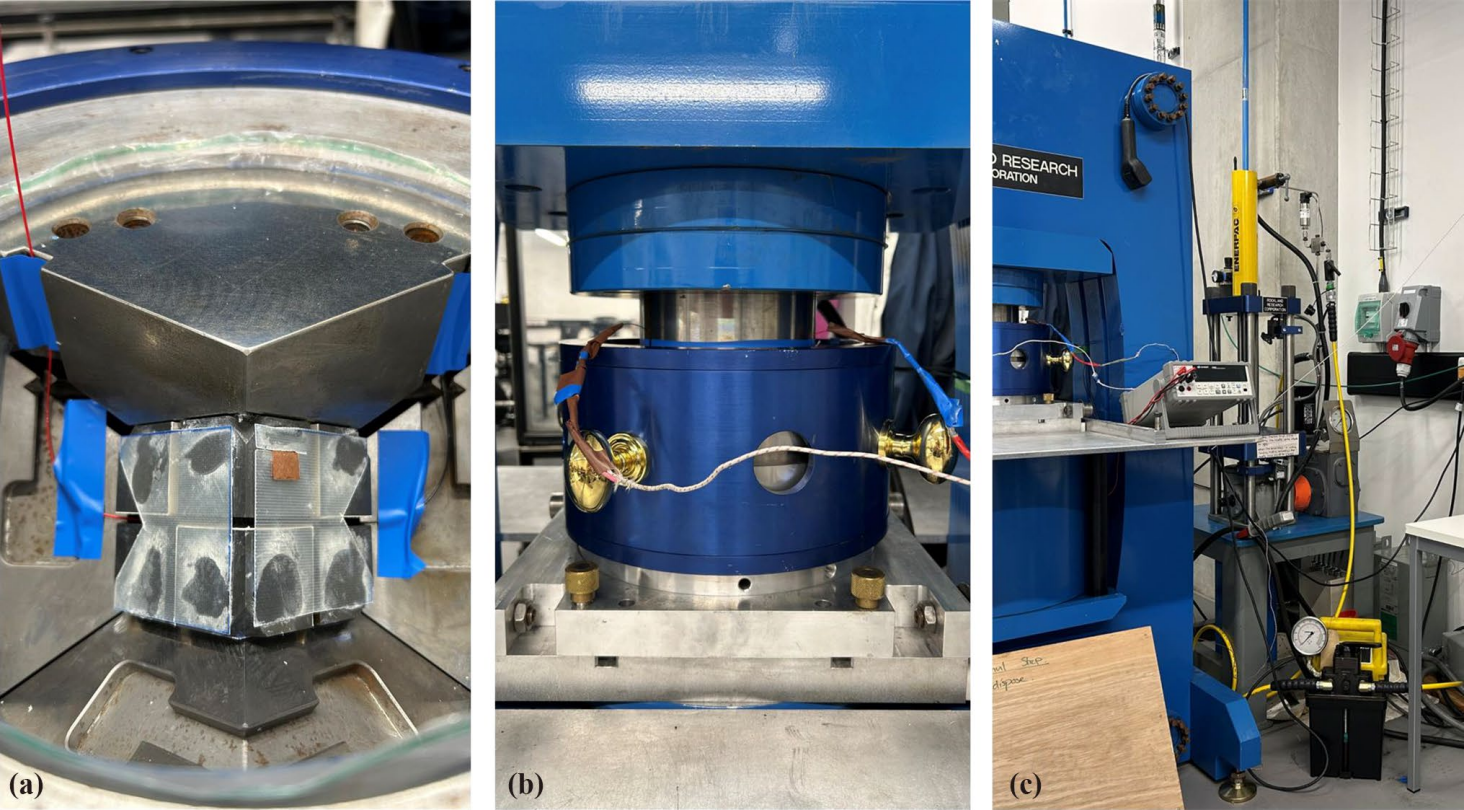
**a** Photograph of the interior of the module. A complete second-stage assembly rests on three square-face-truncated steel wedges at the bottom of the module and a singular steel wedge is shown placed on. **b** Photograph of the module positioned in the 1000-ton Walker-style multi-anvil apparatus. The module is rolled and locked via pins to a bottom pressure plate attached to a hydraulic ram. An upper cylindrical steel pressure plate rests on top of the first-stage assembly and permits a wired connection to the thermocouples to measure temperature of the experiment. **c** Photograph of the frame of 1000-ton Walker-style multi-anvil press and hydraulic system. The Enerpac submerged pump is shown on the floor to the bottom right, while the Enerpac-modified Rockland screw pump is shown in the background.

High P–T experiments were carried out in a 1000-ton Walker-module multi-anvil press [44] (Fig. 2a–c) developed by Rockland Research Corporation in the Department of Materials at the University of Manchester. The octahedron high P cell was surrounded by eight cubic tungsten-carbide (WC) anvils machined to have 11-mm edge length triangular corner truncations, an 18/11 set up, that are positioned against each of the eight faces of the octahedron high P cell (second-stage anvil assembly). Trapezoidal gaskets of pyrophyllite were positioned between each of the WC anvils near the corner truncations, with a small hole drilled into two gaskets to accommodate the extruding thermocouple wires from the high P cell (Fig. 1b). A segment of the thermocouple wires extruding from the high P cell and through the gaskets was tightly wound with thin copper wire (Fig. 1b and c) as a protective measure to maintain electrical contact and therefore T measurements, in case the wires broke at any point during compression and/or heating. Thin sheets of Teflon were glued to each cubic face to ensure the stability of the assembly and provide additional electrical insulation. Cuts in the sheets allowed the wires to extrude from the assembly unimpeded, and small pieces of copper foil were wrapped or folded about small incisions made in the sheets. The copper pieces were positioned against the two WC cubes that have truncations contacting both stainless-steel ends of the high P cell. The second-stage assembly was placed into the module containing a lower set of three stainless-steel wedges with truncated square faces, and a second set of three wedges was placed on top (first-stage anvil assembly) to completely enclose the second-stage assembly in the module (Fig. 2a). A cylindrical steel pressure plate was placed on top of the wedges, and the thermocouple wires were fed through machined gaps in the top set of wedges and pressure plate and sheathed with electrical insulation.

The module was positioned into the frame of the press and locked onto a bottom pressure plate attached to a hydraulic ram (Fig. 2b). The hydraulic ram moved vertically to compress the pressure plate against the wedges that transfers and redirects the applied force to the high P cell, and its motion was controlled by two pumping systems (Fig. 2c): i) an Enerpac submerged pump (PEJ1401E) and ii) an Enerpac-modified Rockland screw pump. The submerged pump was used to quickly apply a small initial hydraulic load within the range of 100–250 psi, which is equivalent to a sample confining P of 0.18–0.44 GPa as determined from our P calibration (Supplementary Information; Fig. S1), while the screw pump was used to apply a slower and more gradual increase of the hydraulic load up to the target sample confining P, as well as maintain the hydraulic load for the entire duration at high T. A high alternating current with was introduced into the P cell graphite furnace via the wedge-copper piece contact to generate high T and held at the target temperature for 5 h. Heating control is provided by a Eurotherm temperature controller and thyristor, which control the voltage supplied to a 40 V/50A step-down transformer, the secondary side of which is connected to the assemblies and furnace and therefore controlled heating.

### Raman spectroscopy and X-ray diffraction

Raman spectra were measured on polished cross-sections of post-experiment samples using a Renishaw InVia Raman Microscope with an excitation laser of wavelength of 633 nm (Renishaw RL633 laser) at the University of Manchester. A high laser power of 9.6 mW was used and kept constant during the measurements, as well as a 20 × objective with a beam diameter of approximately 2 μm. The highest grating of 2400 grooves per mm was used initially to collect the Raman spectra for the LiMnPO_4_ powder and one post-experiment sample. However, since the spectral resolution of the 2400 grating (1.0 cm^−1^) was similar compared to a grating of 1800 grooves per mm (1.2 cm^−1^), the latter grating was used for all other measurements and additionally spanned a larger Raman shift range per measurement. Each Raman spectrum had a 2-s integration time for 40 accumulations, and all spectral measurements were fitted using Gaussian/Lorentzian amplitude functions using Renishaw’s proprietary WiRE (Windows-based Raman Environment) 4.2 software.

Powder XRD patterns were measured from a ground recovered sample and collected using a benchtop Proto AXRD diffractometer having a Bragg–Brentano geometry, an unmonochromated copper radiation source (Cu Kα_1_ λ = 1.540598 Å, Cu Kα_2_ λ = 1.544426 Å) operated at 30 kV with a 20 mA emission current and nickel Kβ absorber (Kβ = 1.392250 Å), and a Dectris Mythen 1 K detector. Continuous scans were recorded for ~ 3.5 h from 10 to 130° 2θ in 2θ increments of 0.0197°. Rietveld refinements were conducted with GSAS-II software, and the fitting was performed from 15 to 130° 2θ with β-LiMnPO_4_ (ICSD Collection Code: 118,216) and was used as the initial atomic and crystallographic structure. Background fitting used a logarithmic interpolation method with nine coefficients. Initial refinement was comprised of only the instrument parameters, background coefficients, and unit cell parameters, and refinement was further improved via sequential introduction of sample displacement and transparency, microstrain, crystallite size, spherical harmonic-preferred orientation model, and atomic position as parameters. A total of 58 parameters were used in the refinement of 5843 data points and uncertainties associated with the modelled fit.

## Results and discussion

### Powder X-ray diffraction

**Figure 3:**
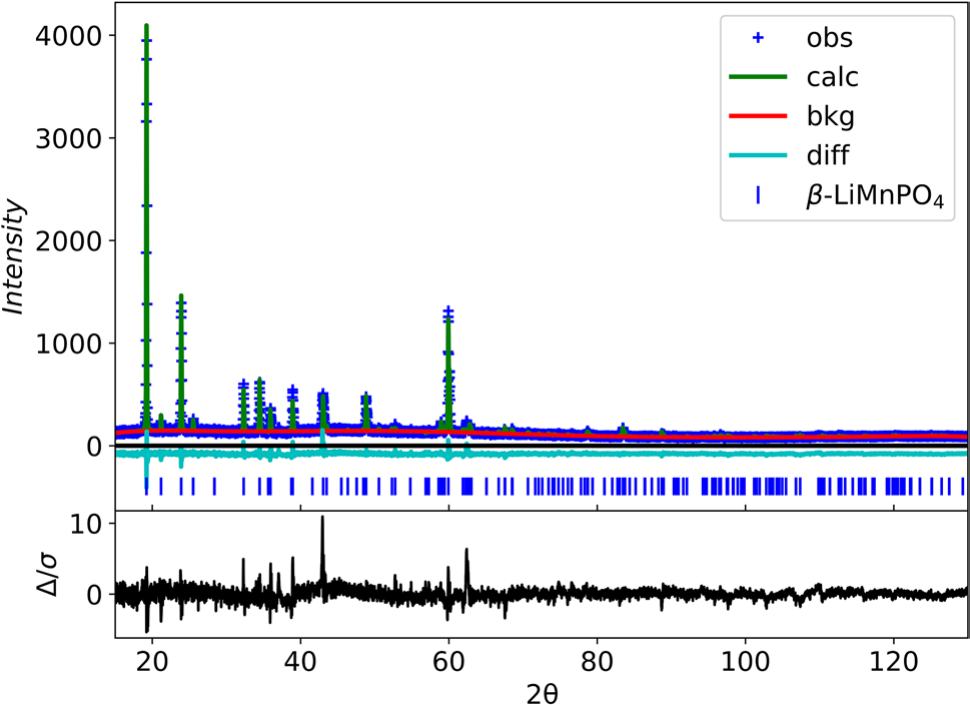
Powder X-ray diffraction pattern of LiMnPO_4_ recovered after a hot-pressing experiment conducted at 8 GPa and 938 °C using Rietveld refinement and GSAS-II software: observations (blue cross), background model (red line), calculated model (green line), residuals (cyan line), and Bragg reflections (blue dash). ∆/σ represents the residuals normalised to the standard deviation. The results show the *Cmcm* structure accounts for all diffraction peaks, indicating that β-LiMnPO_4_ was synthesised.

Figure 3 shows the XRD pattern between 15 and 130° 2θ of the sample recovered from 8 GPa and 938 °C, and the results of the Rietveld refinement determined the structure of the high P LiMnPO_4_ phase to be the β phase. The *Cmcm* β phase accounts for all the peaks in the diffraction pattern, and Table 1 shows that the unit cell parameters of our hot-pressed β-LiMnPO_4_ are in good agreement with the solvothermal β-LiMnPO_4_ of Assat and Manthiram [43]. Apparent inconsistencies between the between the calculated model and observed intensities, such as the mismatch at ~ 43° 2θ revealed by normalised residuals, are due to the windows of the stage of the diffractometer [30]. Moreover, our determined high P phase of LiMnPO_4_ is the same high P *Cmcm* structure reported for LiFePO_4_, LiNiPO_4_, and LiCoPO_4_ [36–38]. The solvothermal synthesised sample exhibited a smaller cell volume than our recovered sample with only the *c* axis being larger, and these differences may be due to different synthesis techniques.

**Table 1:** Crystallographic data of α (*Pnma*) and β (*Cmcm*) structures of LiMnPO_4_. Our high-pressure–temperature hot-pressing experimental results are compared to lower pressure–temperature solvothermal synthesis results [43]. Our results of the starting α-LiMnPO_4_ (Supplementary Information; Fig. S2) are also compared to prior works [45]. **Li et al. [45] report the orthorhombic α-LiMnPO_4_ using the *Pnmb* space group. *Pnmb* is isomorphic with the *Pnma* space group, and this table transposes their results to *Pnma*

β-LiMnPO_4_			α-LiMnPO_4_	
This work		Assat and Manthiram [43]	This work	Li et al. [45]**
*a* (Å)	5.5465(3)	5.5312(3)	10.466(2)	10.452(1)
*b* (Å)	8.4110(6)	8.3988(3)	6.118(1)	6.106(1)
*c* (Å)	6.2990(4)	6.3160(4)	4.755(1)	4.746(1)
*V* (Å^3^)	293.87(6)	293.41(2)	304.4(2)	302.9
*R*_w_ (%)	7.46	14.3	17.23	
*χ*^2^	0.85	1.62	1.60	

### Raman spectroscopy

**Figure 4:**
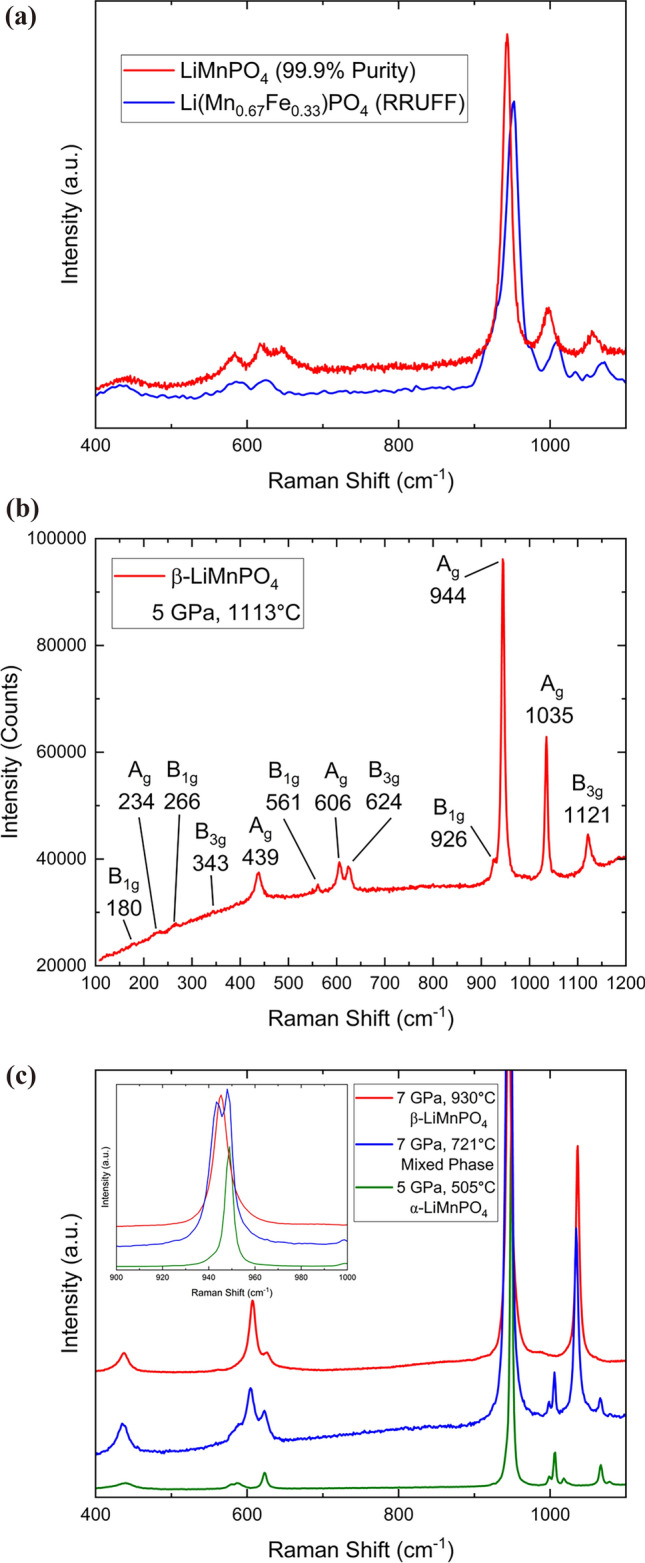
**a** Raman spectra of our starting α-LiMnPO_4_ powder compared to naturally occurring lithiophilite (α-Li(Mn,Fe)PO_4_) obtained from the RRUFF Comprehensive Database of Mineral Data (RRUFF ID: R070303). **b** Raman spectrum and assigned phonon symmetries [48] of β-LiMnPO4 recovered from an experiment conducted at 5 GPa and 1113 °C. **c** Raman spectra from our recovered experiments comparing α, mixed (α + β), and β phases of LiMnPO_4_.

Raman spectra of naturally occurring Lhp have been reported [46], and the Raman shift peak positions of our α-LiMnPO_4_ powder starting material are compared to Lhp, as shown in Fig. 4a. The slight differences may be attributed to the chemical composition of naturally occurring Lhp typically being a Mn-rich intermediate member of the Trp-Lhp solid solution series, alongside other impurities of lighter metals such as calcium and magnesium [46, 47]. Regardless, our measured results for the starting material and high P–T α phase are in good agreement with natural Lhp.

Figure 4b shows the Raman spectra of β-LiMnPO_4_ of a recovered sample that was compressed and heated to 5 GPa and 1113 °C. Using the results of their ab initio simulations, Bandiello et al. [48] assigned phonon symmetry to the Raman modes of the β phase of LiNiPO_4_ and of the theoretical 18 Raman-active modes; they were able to experimentally measure 14 at ambient conditions. Since the chemistry and Raman shifts are comparable and the solid-state structure is the same, we adopted the same phonon symmetry assignments to the Raman modes of β-LiMnPO_4_ and measured at least 12 Raman-active modes. To complement Fig. 4c, Table 2 includes a list of all assignments for the three experiments that completely synthesised the high P phase. As shown, not all Raman active modes could be assigned and/or measured because of weak intensities or their absence, and, in the case of our experiment conducted at 7 GPa, active modes were outside the range of our spectroscopic analysis. Bandiello et al. [48] noted a similar disappearance of Raman modes relative to ambient conditions, while the sample was compressed to high P in a diamond anvil cell as they were able to measure in situ only 9 of the 14 previously measured modes. The modes not measured in situ at high P or, in our case, ex situ at ambient conditions were predominantly modes with weak intensities at lower Raman shift values that are attributed to vibrations of Li^1+^, which are active in the β phase but not in the α phase [48].

**Table 2:** Experimentally measured Raman shift values of prominent peaks appearing in the Raman spectra of our synthesised β-LiMnPO_4_ and β-LiNiPO_4_ [48] alongside 18 theoretical Raman active mode symmetries. Samples recovered from our 5 and 8 GPa experiments were analysed with an 1800 grating, while the sample recovered from our 7 GPa experiment was analysed with a 2400 grating and had a reduced measurement range (empty indicates outside range). Measurement uncertainties of the Raman shift are < 2 cm^−1^

Mode symmetry	Raman Shift (cm^−1^)			
	LiMnPO_4_		LiNiPO_4_	
	5 GPa, 1113 °C	7 GPa, 930 °C	8 GPa, 938 °C	Bandiello et al. [48]
B_3g_	–		–	–
B_1g_	180		180	185
B_1g_	–		–	–
A_g_	234		226	250
B_1g_	266		262	259
B_2g_	–		–	–
B_3g_	343		–	343
A_g_	–		–	–
B_2g_	–	–	–	444
A_g_	439	438	437	454
B_3g_	–	–	–	497
B_1g_	561	–	561	565
A_g_	606	607	607	619
B_3g_	624	626	624	647
B_1g_	926	–	926	912
A_g_	944	945	944	941
A_g_	1035	1036	1034	1058
B_3g_	1121		1120	1147

Figure 4c includes a comparison between the Raman spectra of the α and β phases measured from two recovered samples from experiments that were conducted at P–T conditions of 5 GPa and 505 °C and 7 GPa and 930 °C, respectively. The difference in the Raman shifts of prominent peaks is shown to be substantial enough to differentiate between the phases, including mixtures of α and β phases, and provided clear constraints for the α-β phase boundary. Furthermore, no thermal decomposition and/or a phase transition in LiMnPO_4_ due to exposure of the sample to the Raman laser was observed, as demonstrated by a controlled experiment measuring Raman spectra as a function of laser power (Figure S3).

### Pressure–temperature phase diagram

**Figure 5:**
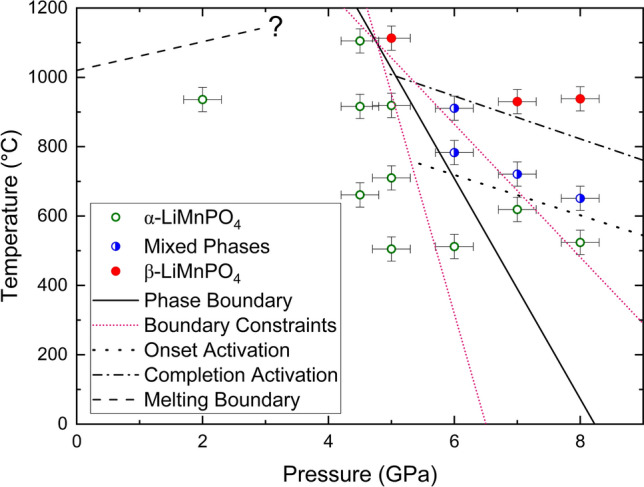
Derived pressure–temperature phase diagram. The green unfilled, blue half-filled, and red filled circles represent α-, mixed (α + β), and β phases of LiMnPO_4_ determined using Raman spectroscopy and the uncertainties on pressure and temperature are ± 0.3 GPa and ± 35 °C. The solid black linear phase boundary line was delineated based on Raman spectra results and consideration for reaction kinetics. The pink stippled lines represent maximum and minimum phase boundary constraints with respect to the slope values of the line. To the right of the phase boundary: i) below the black dotted line is the region with inhibited reaction kinetics to initiate the α → β transition; ii) between the black dotted and dash-dot lines is the region with insufficient reaction kinetics to complete the α → β transition; iii) above the black dash-dot line is the region where the α → β transition has reached completion. The positive-pressure dependency of the dashed melting boundary extended from the average of reported melting temperatures at 1 atm pressure [55–57] is inferred from melting not having occurred during our experiments.

Figure 5 shows the P–T phase diagram of LiMnPO_4_ up to 9 GPa and 1200 °C based on measured α-, mixed, and β-phases of samples recovered from hot-pressing synthesis experiments. A mixed phase structure is indicated by the observation of prominent Raman peak shifts associated with both α- and β-structures in the Raman spectra (Fig. 4c). The measured phases, alongside consideration for reaction kinetics, were used to delineate the onset of the α-β phase transition. The negative slope of the phase boundary, coupled with the negative volume change (Table 1), indicated a negative latent heat of reaction term. The Clapeyron relation was determined to be -3.15 $\genfrac{}{}{0pt}{}{\mathrm{m}\mathrm{a}\mathrm{x}=-1.58}{\mathrm{m}\mathrm{i}\mathrm{n}=-5.22}$ MPa/°C, where the sub- and super-script values are the linear boundary constraints. The linear phase boundary and constraints assumed a point between the two highest T (~ 1100 °C) experiments conducted at 4.5 GPa (α-LiMnPO_4_) and 5 GPa (β-LiMnPO_4_) where all lines intersect. In addition to the intersection point, the shallowest boundary constraint line in Fig. 5 passes through the mixed phase at 6 GPa and 783 °C, the lowest P–T conditions observed for this phase, within the uncertainties, thus representing an upper bound of the phase boundary. Similarly, the steepest boundary constraint line considered the two α-phases measured at 5 GPa and 710 °C and 5 GPa and 919 °C, two results that are closest to and at T conditions comparable to or exceeding both mixed and β phases at higher P and thus represent a lower bound.

A mixed phase region was only observed at higher experimental P (6–8 GPa), suggesting either (a) the run time was not sufficient to provide the energy that drives the kinetics of the α → β transition to completion, a phenomena previously observed in, for example, SiO_2_ [49] and MgGeO_3_ [50], or (b) the volume change buffered the phase reaction, as reported for other systems at high P–T conditions [51–54], or a combination of the two. On the right side of the phase boundary line, the regions between the dotted onset activation line and dash-dot completion activation line are P–T conditions that are not sufficient to drive the α → β transition to completion and the regions between the phase boundary and the dotted onset line are P–T conditions that are not sufficient to initiate the α → β transition.

In good agreement with our predicted room T α → β-LiMnPO_4_ at ~ 8.2 GPa, Bandiello et al. [48] calculated an α → β transition of LiNiPO_4_ at 8 GPa. However, after compressing α-LiNiPO_4_ at room T, the β phase was not observed until ~ 41 GPa, which they attributed to a high kinetic barrier as observed in other phosphates. Moreover, at high T (600 °C), they achieved a complete transformation to the β-LiNiPO_4_ at 6.5 GPa. These experimental and calculational results showed an inhibition of α → β transformation kinetics and negative Clapeyron relation comparable to our results. Similarly, while only α-LiFePO_4_ was observed up to 21.5 GPa at room T [55], a complete transformation to β-LiFePO_4_ was achieved at 6.5 GPa and 900 °C [37]. As well, α-LiCoPO_4_ underwent a complete α → β transformation at 6 GPa and 900 °C [36]. According to the phase boundary line in Fig. 5, a α → β transition of LiMnPO_4_ at 6 GPa will occur at > 705 °C, and this result is consistent with the high P–T behaviour of the aforementioned LiMPO_4_ compounds.

Previously reported melting T of α-LiMnPO_4_ at ambient P range from 1001 to 1040 °C [56–58]. Our equilibrium phase boundary line, including the boundary constraints, intersects ambient P at significantly greater T than the observed ambient P melting T, and therefore, β-LiMnPO_4_ is not a stable phase at ambient P. Our highest T experiments conducted at 4.5 GPa (1105 °C) and 5 GPa (1113 °C) are greater than melting at ambient P but showed no evidence of melting. Our results suggest a positive P-dependent melting boundary, consistent with other olivine group minerals [59–61]. Lastly, our results suggest that Assat and Manthiram [43] were able to synthesise the high P–T β-LiMnPO_4_ phase at significantly lower P–T conditions, albeit metastable. As such, the solvothermal method could provide a reliable alternative pathway to synthesise β-LiMnPO_4_ for further study of its physicochemical properties in lieu of high P apparatuses. While the previous work noted a sluggish reversion to the α phase with a relatively small increase in T above their 275 °C synthesis T, the effect of increasing P on the stability of this metastable state remains unclear. In addition to determining the stability of this metastable state, in situ XRD of LiMnPO_4_ at high P–T conditions will be needed to determine thermodynamical properties and equations of state of the α- and β-LiMnPO_4_ phases.

## Conclusion

A solid-state phase boundary of the potential candidate cathode material LiMnPO_4_ was experimentally determined at pressures up to 8 GPa and temperatures up to 1113 °C. The high-pressure–temperature phase boundary was delineated using Raman spectroscopy, and the crystal structure was determined using X-ray diffraction and Rietveld refinement. The boundary separates the ambient condition olivine *Pmna* orthorhombic (α) structure from a high-pressure *Cmcm* orthorhombic (β) structure (*a* = 5.5465(3) Å; *b* = 8.4110(6) Å; *c* = 6.2990(4) Å). A linear Clapeyron relation was determined to be -3.15 MPa/°C and predicts a room-temperature α → β transition at ~ 8.2 GPa. This work complements previous synthesis methods that produced metastable β-LiMnPO_4_ by confirming β is the high-pressure structure and producing the first phase diagram describing the stability field of the electrochemically active α and inactive β structures in P–T space. Moreover, our results show that LiMnPO_4_ behaves like other cathode materials, LiFePO_4_, LiNiPO_4_, and LiCoPO_4_, which undergo an α → β transition at high P–T.

## Supplementary Information

Below is the link to the electronic supplementary material.

Supplementary file1 (DOCX 674 kb)

## Data Availability

All data generated or analysed during this study are included in this published article and its supplementary information files. Additionally, a crystallographic information file (.cif) containing all crystallographic information of the β-LiMnPO_4_ is available in the Figshare repository, 10.48420/32186136.v1.
