# Supplementary material for: Determination of the high-pressure–temperature phase of LiMnPO4 energised by battery applications
Source: J Mater Sci. 2026 Jun 7;61(28):20189–201. doi: 10.1007/s10853-026-13008-z (PMC13264576; doi:10.1007/s10853-026-13008-z)
Supplement: Supplementary file 1 — Supplementary file1 (DOCX 674 kb) [file 10853_2026_13008_MOESM1_ESM.docx]

*Supplementary Information*

Journal of Materials Science

**Determination of the High Pressure-Temperature Phase of LiMnPO_4_ Energized by Battery Applications**

Joshua A.H. Littleton^a*^, Andrew J.M. Evans^a,b^, Julia Neukampf^b^, Tara R. McElhinney^a^ and Simon A. Hunt^a*^

^a^Department of Materials, University of Manchester, Manchester, M13 9PL, UK

^b^Department of Earth and Environmental Sciences, University of Manchester, Manchester, M13 9PY, UK

* Corresponding Authors, josh.littleton@manchester.ac.uk; simon.hunt@manchester.ac.uk

Contents:

Figure S1: Pressure calibration of a Walker-style multi-anvil apparatus at room temperature.

Figure S2: X-ray diffraction of purchased LiMnPO_4_ powder starting material.

Figure S3: Raman spectra of LiMnPO_4_ as a function of laser power.

Table S1: Atomic positions and symmetries of β-LiMnPO_4_.

References





**Figure S1:** Results of a room temperature pressure calibration experiment showing confining pressure as a function of oil pressure for an 18/11 setup in a Walker-style multi-anvil apparatus. The pressure calibration monitored *in-situ* the electrical resistance of bismuth (Bi). At room temperature, Bi undergoes I-II, II-III, and III-V solid-state phase transitions (solid green circles) at approximately 2.5, 2.7, and 7.7 GPa [1-2], respectively, that are each accompanied by electronic transitions resulting in changes of the electrical resistance. The calibration curve ends at 8800 psi, which is the maximum oil pressure of the hydraulic system.


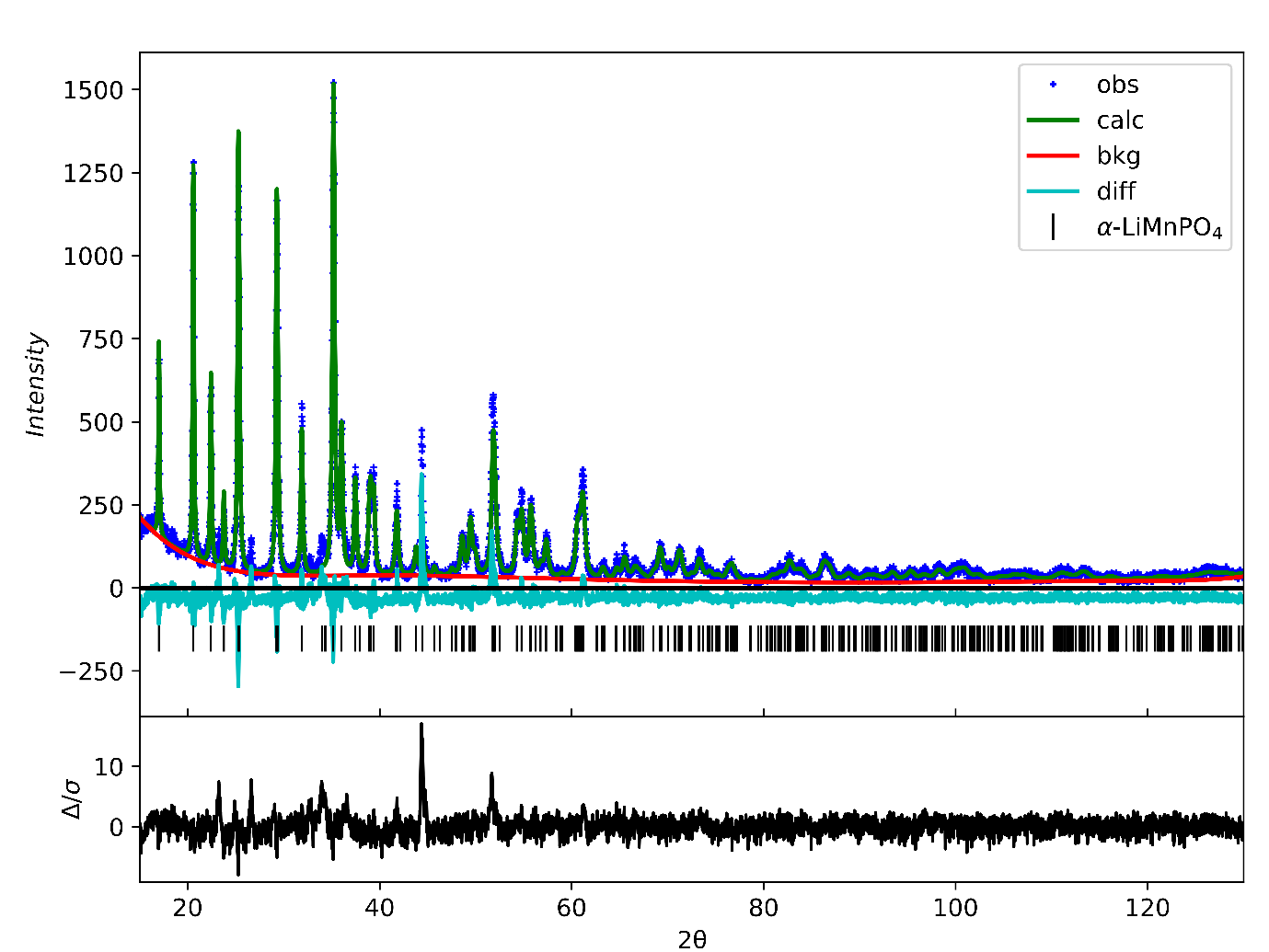


**Figure S2:** X-ray diffraction (XRD) results of the starting sample material, LiMnPO_4,_ showing the purchased powder has an olivine *Pmna* structure (α). Powder XRD patterns were measured and collected using a Bruker D8 Discover diffractometer having a Bragg-Brentano geometry, an unmonochromated copper radiation source (Cu Kα_1_ λ = 1.540594 Å, Cu Kα_2_ λ = 1.544408 Å) and a Lynxeye XE-T detector. Continuous scans were recorded for ~1.4 hours from 10-130° 2θ in 2θ increments of 0.02°. Rietveld refinements were conducted with GSAS-II software: observations (blue cross), background model (red line), calculated model (green line), residuals (cyan line), and Bragg reflections (black dash). ∆/σ represents the residuals normalised to the standard deviation. The fitting was performed from 15-130° 2θ with α-LiMnPO_4_ (ICSD Collection Code: 214712) used as the initial atomic and crystallographic structure. Background fitting used a logarithmic interpolation method with nine coefficients. Initial refinement comprised of only the background coefficients and unit cell parameters, and refinement was further improved via sequential introduction of sample displacement and transparency, microstrain, spherical harmonic preferred orientation model, and atomic position as parameters. A total of 36 parameters were used in the refinement of 5637 data points.


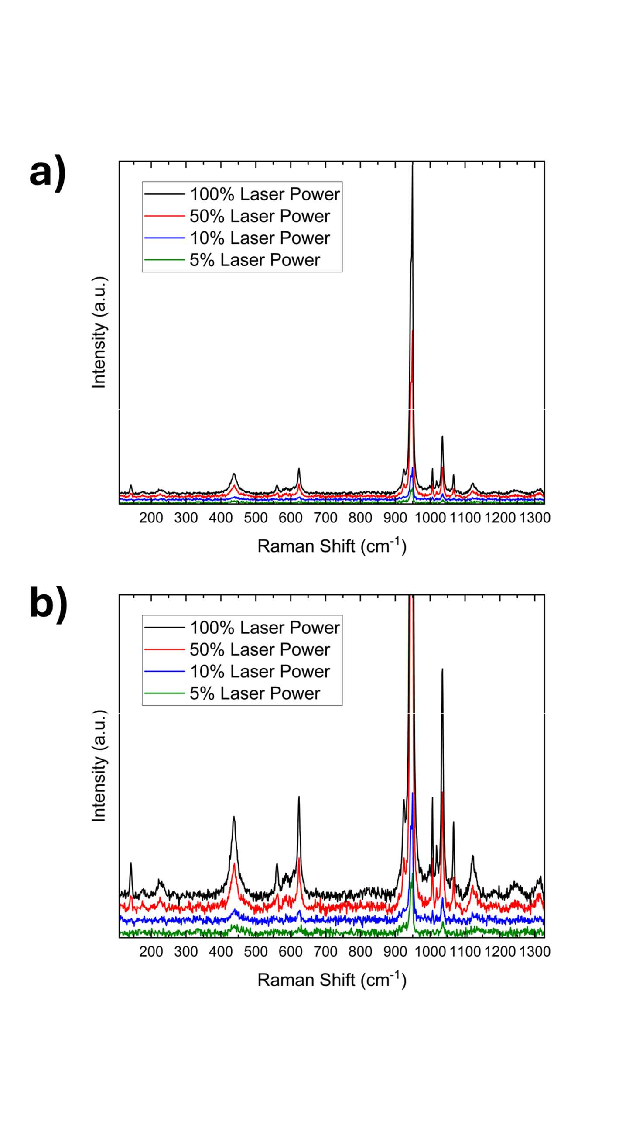


**Figure S3:** a) Raman spectra of mixed (α+β) phase LiMnPO_4_ as a function of laser power measured using a Renishaw InVia Raman Microscope with an excitation laser of wavelength of 633 nm (Renishaw RL633 laser), 20x objective with a beam diameter of ~2 μm, and grating of 1800 grooves per mm. b) The same Raman spectra as in a) with the scale adjusted to focus on less intense Raman shift peaks. The laser powers used were: 5% (0.47 mW), 10% (0.97 mW), 50% (4.8 mW), and 100% (9.6 mW). The signal-to-noise ratio increased with increasing laser power. The absence of new Raman shift peaks with increasing laser power shows that thermal decomposition and/or a phase transition in LiMnPO_4_ due to exposure to the laser did not occur.

**Table S1:** Post-Rietveld refinement atomic positions of the high pressure-temperature β-LiMnPO_4_ (*Cmcm*) crystallographic structure using GSAS-II.

| **Atomic Species** | **Wyckoff Positions** | **x/a** | **y/b** | **z/c** | **Occupancy Fraction** | **Isotropic Atomic Displacement (U_iso_)** |
| --- | --- | --- | --- | --- | --- | --- |
| Li | 4c | 0.000000 | 0.700(6) | 0.250000 | 1.000 | 0.0147 |
| Mn | 4a | 0.000000 | 0.000000 | 0.000000 | 1.000 | 0.0120 |
| P | 4c | 0.000000 | 0.3272(9) | 0.250000 | 1.000 | 0.0137 |
| O_1_ | 8f | 0.000000 | 0.2649(13) | 0.058(4) | 1.000 | 0.0048 |
| O_2_ | 8g | 0.2280(15) | 0.4206(8) | 0.250000 | 1.000 | 0.0048 |

**References**

[1] Chen H, Xiang S, Yan X, et al (2016). Phase transition of solid bismuth under high pressure. Chin. Phys. B. 25: 108103. <https://doi.org/10.1088/1674-1056/25/10/108103>

[2] Ono S (2018) High-pressure phase transition of bismuth. High Pressure Res. 38: 414-421. <https://doi.org/10.1080/08957959.2018.1541456>
